# From shadows to clarity: A new paradigm for comprehensive variant detection in undiagnosed dystrophinopathy using combined long-read and RNA sequencing

**DOI:** 10.1016/j.csbj.2025.10.049

**Published:** 2025-10-24

**Authors:** Lei Zhao, Shirang Pan, Chaoping Hu, Yiyun Shi, Xihua Li

**Affiliations:** a Department of Neurology, Children's Hospital of Fudan University, Shanghai, China; b Grandomics Biosciences, Beijing, China

**Keywords:** Dystrophinopathy, Duchenne muscular dystrophy, Muscle biopsy, Long-read sequencing, RNA-seq, Pseudoexon, Deep intronic variants

## Abstract

Approximately 2–5 % of dystrophinopathy cases remain undiagnosed at the molecular level following standard multiplex ligation-dependent probe amplification (MLPA) and exome sequencing (ES), precluding these patients from variant-specific genetic counseling and therapy eligibility assessment. We developed an integrated diagnostic pipeline combining targeted long-read sequencing (LRS) with muscle RNA-seq and applied it to 50 Chinese patients (46 males, 4 females) with biopsy-confirmed dystrophinopathy in whom MLPA and ES had yielded negative results. A molecular diagnosis was achieved for all 50 probands: 30 (60 %) harbored intronic single nucleotide variants (SNVs) leading to pseudoexon inclusion or exon skipping, while the remaining 20 (40 %) carried complex structural variants (SVs), including inversions (n = 13), translocations (n = 5), and insertions/deletions (n = 2). Of these, 23 (46 %) were novel variants not previously recorded in disease databases, and RNA-seq confirmed their aberrant splicing effects. A notable discovery was a 1.9 kb intronic inversion that uniquely activated cryptic splice sites on the antisense strand, representing a previously unreported pathogenic mechanism in dystrophinopathies. Cascade screening identified 45 carriers across 28 families, revealing a maternal carrier rate of 84.8 % (28/33). This integrated LRS and RNA-seq approach demonstrates significant value in resolving molecularly undiagnosed dystrophinopathy cases and enabling comprehensive genetic counseling. Furthermore, it establishes a robust diagnostic paradigm for molecularly unresolved neuromuscular disorders.

## Highlights

• Combined LRS and RNA-seq resolve 100 % MLPA/ES-undiagnosed dystrophinopathy cases.
• Twenty-three novel pathogenic variants were detected by the integrated approach.
• Underrecognized pathogenic mechanism: Intronic inversion variant activates cryptic splice sites on the antisense strand.
• Precise mapping of complex SVs and deep intronic SNVs enables evidence-based carrier screening and genetic counseling.

## Graphical Abstract

**Figure:**
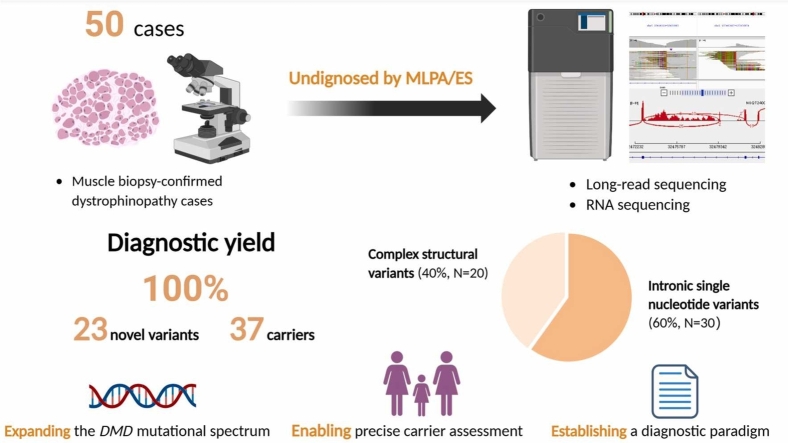

## 1 Introduction

Dystrophinopathies represent a spectrum of X-linked neuromuscular disorders arising from pathogenic variants in the *DMD* gene (Xp21.2; OMIM #300377). This gene encodes dystrophin, a cytoskeletal protein critical for maintaining muscle membrane stability [1], [2], [3]. The clinical manifestations encompass four primary phenotypes: Duchenne muscular dystrophy (DMD), Becker muscular dystrophy (BMD), X-linked dilated cardiomyopathy (XLDCM), and symptomatic manifestations in female carriers. In males, hemizygous loss-of-function variants in the *DMD* gene are responsible for over 99 % of cases. Females are typically asymptomatic carriers; however, symptomatic disease can occur due to skewed X-chromosome inactivation or significant chromosomal rearrangements [4], [5]. Reported incidence rates of skeletal muscle damage among female carriers (including those who are asymptomatic) range from 2.5 % to 19 %, while the prevalence of dilated cardiomyopathy is 7.3 %–16.7 % in carriers of DMD-causing variants and 0 %–13.3 % in carriers of BMD-causing variants [4].

Pathogenic *DMD* variants are conventionally categorized into three groups: (1) large deletions or duplications (70–80 %), detectable by multiplex ligation-dependent probe amplification (MLPA); (2) small insertions/deletions or single nucleotide variants (SNVs) (18–20 %), identifiable through exome sequencing (ES); and (3) complex structural variations (SVs) or deep intronic splice-altering variants (2–5 %), which often escape detection by standard methods [6], [7], [8], [9]. Each of these categories presents specific challenges for conventional diagnostics. Although MLPA and ES serve as first-line tests, their limitations are well-recognized: MLPA is susceptible to false-positive single-exon deletions caused by probe hybridization artifacts, while ES lacks coverage of non-coding regions that may harbor cryptic splice sites or complex SVs. As a result, approximately 2–5 % of patients remain without a molecular diagnosis even after comprehensive testing, highlighting a critical need for more advanced diagnostic strategies [6], [10], [11]. To address this diagnostic gap, we hypothesized that a combined approach using long-read sequencing (LRS) and RNA sequencing (RNA-seq) could identify the elusive variants in these unresolved cases.

Recent advances have highlighted the role of non-canonical splicing defects as major contributors to molecularly undiagnosed dystrophinopathies. Deep intronic variants can activate cryptic splice sites, leading to pseudoexon generation, while SVs, such as inversions and translocations, may disrupt splicing regulatory elements or alter higher-order gene architecture [12], [13]. Although RNA-seq of muscle biopsies can detect aberrant splicing events, its diagnostic utility remains limited by the invasiveness of the biopsy procedure and the difficulty in linking transcript-level findings to underlying DNA variants [12], [14]. The emergence of LRS technologies now allows comprehensive detection of SVs and haplotype-phasing of complex rearrangements across the extensive 2.2-Mb *DMD* locus [7]. When integrated with RNA-seq, this combined approach effectively bridges the gap between DNA-level variation and transcriptomic outcomes, establishing a unified framework for molecular diagnosis.

In this study, we evaluated the diagnostic yield of an integrated approach combining long-read sequencing of the entire *DMD* gene with RNA-seq in a cohort of 50 dystrophinopathy patients who had remained undiagnosed following standard MLPA and ES. To our knowledge, this represents the largest cohort of molecularly unresolved cases of this disorder reported to date. Our methodology achieved a 100 % diagnostic success rate within this cohort, resolved complex structural variants with nucleotide-level precision, and identified multiple novel deep intronic variants responsible for splicing dysregulation, thereby confirming their pathogenicity. These findings establish a robust diagnostic paradigm for molecularly undiagnosed neuromuscular disorders. Furthermore, this approach holds potential for extension to other genetic diseases involving elusive non-coding variants, although further validation in diverse disease cohorts is warranted.

## 2 Methods

### 2.1 Study cohort

This cohort study was approved by the Ethics Committee of the Children's Hospital of Fudan University (Approval No. [2022]300 A). Between April 2014 and December 2023, 50 unrelated Chinese probands with muscle biopsy-confirmed dystrophinopathy were enrolled, comprising 46 males (92 %) and 4 females (8 %), with a median age at diagnosis of 5.1 years (range: 1–13.2 years). All participants had previously tested negative for pathogenic variants using both MLPA and ES; of whom 48 had no pathogenic variants detected, and 2 carried variants of uncertain significance (VUS). Each patient presented with clinical hallmarks of dystrophinopathy, such as progressive muscle weakness and elevated serum creatine kinase levels, or had a family history indicative of X-linked muscular dystrophy. After ethical approval, these probands were enrolled for further genetic analysis: 44 diagnosed between April 2014 and December 2022 were contacted and enrolled after obtaining written informed consent from patients or their legal guardians, while 6 newly diagnosed between January 2023 and December 2023 were enrolled with consent at diagnosis. Detailed clinical characteristics are summarized in Supplementary Table S1 and Supplementary Figure S1. Additionally, voluntary written informed consent was obtained from extended relatives who underwent cascade testing.

### 2.2 Muscle biopsy and immunohistochemistry

Open surgical muscle biopsies were obtained from the biceps brachii under local anesthesia. Fresh tissue specimens were immediately snap-frozen in isopentane pre-cooled with liquid nitrogen. Serial 8-μm transverse cryosections were prepared for subsequent analysis. Histopathological evaluation was performed using hematoxylin and eosin (H&E) staining. Immunohistochemical analysis was conducted using a panel of anti-dystrophin monoclonal antibodies (Leica Biosystems, Germany) targeting distinct protein domains: NCL-DYS1 (rod domain), NCL-DYS2 (C-terminus), and NCL-DYS3 (N-terminus). The antibodies were applied at the following optimal working dilutions: NCL-DYS1 at 1:50, and both NCL-DYS2 and NCL-DYS3 at 1:20.

### 2.3 Nucleic acid extraction

Genomic DNA was isolated from peripheral blood leukocytes obtained from all probands and available family members using the CWBIO Blood DNA Kit (CW2361S, CWBIO, China). Total RNA was extracted from approximately 30 mg of snap-frozen muscle biopsy tissue with the RNAprep Pure Tissue Kit (Tiangen Biotech, Beijing, China). All RNA samples subsequently underwent DNase I treatment to remove genomic DNA contamination, and their quality was verified using an Agilent 2100 Bioanalyzer, confirming an RNA Integrity Number greater than 7.0 for all specimens.

### 2.4 Long-read sequencing and variant calling

Targeted long-read sequencing was performed following an established protocol [7]. Briefly, the purified target DNA fragments were amplified by PCR and sequenced on the PacBio Revio platform according to the manufacturer's instructions. For data analysis, the generated long reads were aligned to the reference genome GRCh37/hg19 using minimap2 (v2.24-r1122). SVs and SNVs were subsequently called using Sniffles2 (v2.0.7) and Pepper_DeepVariant (r0.7), respectively.

### 2.5 RNA sequencing and splicing analysis

RNA sequencing was performed on muscle biopsy-derived RNA from patients harboring novel deep intronic variants or SVs. For library preparation, poly(A)+ mRNA was enriched via oligo(dT) selection. Sequencing libraries were constructed using the NEBNext Ultra II Directional RNA Library Prep Kit (#E7420, NEB, USA) and sequenced on an Illumina NovaSeq Xplus platform (Illumina, Inc.), generating approximately 100 million paired-end reads per sample. Variant calling from RNA-seq data was performed using Mutect2 within the GATK4 framework (v4.2.0.0).

The splicing consequences of candidate splice-altering variants were predicted using two complementary tools: the Rare Disease Data Center (RDDC) online RNA Splicer (https://rddc.tsinghua-gd.org/zh/tool/rna-splicer) and SpliceAI (https://spliceailookup.broadinstitute.org).

### 2.6 Statistical analysis

We assessed whether the observed carrier rate among the 33 mothers of affected probands in our cohort significantly differed from the previously reported rate of 64.3 % [9]. A two-tailed binomial test was performed using IBM SPSS Statistics 25 (α = 0.05). The null hypothesis (*H₀*) stated that the true carrier rate (*p*) was equal to the reference value (*p₀* = 0.643), while the alternative hypothesis (*H₁*) posited a difference (*p* ≠ *p₀*). Due to the limited sample size (n = 33), exact binomial probabilities were calculated using the legacy binomial test function in SPSS to ensure accuracy, circumventing potential inaccuracies from asymptotic approximations. A *p*-value less than 0.05 was considered statistically significant, leading to the rejection of *H₀*. Given the small sample, results should be interpreted with caution.

## 3 Results

### 3.1 Diagnostic yield and variant spectrum

**Fig. 1:**
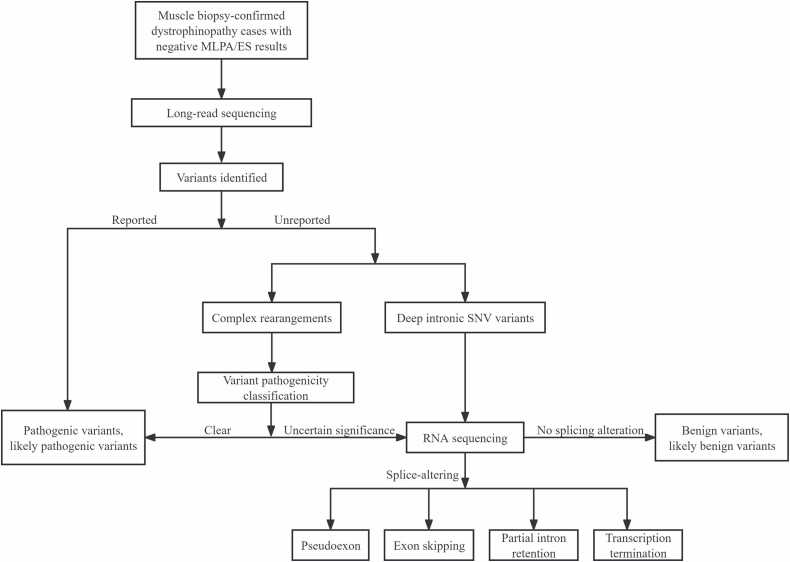
Schematic of the integrated diagnostic pipeline combining LRS and RNA sequencing for comprehensive variant detection in dystrophinopathy.

The integration of LRS and RNA-seq successfully resolved the molecular diagnosis in all 50 dystrophinopathy cases that had remained undiagnosed following standard MLPA and ES, achieving a 100 % diagnostic yield. Across the cohort, we identified a total of 43 unique pathogenic variants. These comprised 30 deep intronic SNVs, accounting for 60 % (30/50) of cases and including 4 novel pathogenic SNVs, and 20 SVs, accounting for the remaining 40 % (20/50). The SVs were further classified into three categories: deep intronic insertions/deletions (n = 2), large inversions (n = 13), and translocations (n = 5). All identified variants were distributed across the entire *DMD* locus. The most frequently observed variant was c.9225–647 A>G (identified in 4 cases), followed by c.9163 + 2510 G>A (in 3 cases). Two variants, c.7661–1646 C>G and c.5448 + 67 A>G, were each observed in two cases, while the remaining 39 unique variants were each identified in a single case (Fig. 1; Tables S2–S3).

### 3.2 Impact of deep intronic variants on splicing

#### 3.2.1 Pseudoexon inclusion

**Fig. 2:**
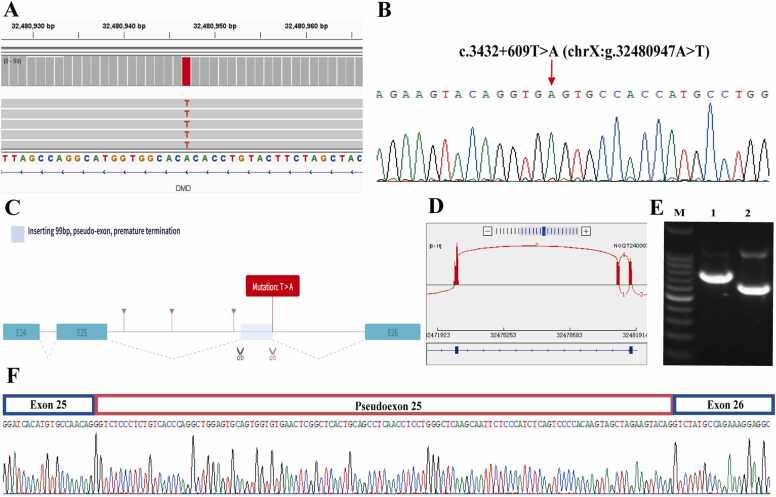
Characterization of the c.3432 + 609 T > A variant leading to pseudoexon inclusion in Patient 14 (P14). (A) Long-read sequencing identifies the single-nucleotide variant c.3432 + 609 T > A (chrX:g.32480947 A>T) in genomic DNA. (B) Sanger sequencing confirms the presence of the c.3432 + 609 T > A variant. (C) Prediction by the RNA Splicer tool indicates an aberrant splicing pattern induced by the deep intronic variant. (D) Sashimi plot from RNA-seq data illustrates the inclusion of a pseudoexon in P14. (E) RT-PCR analysis of exons 22–28 reveals an abnormal fragment in P14 (lane 1), not observed in the healthy control (lane 2). M, molecular weight marker. (F) Schematic representation of pseudoexon 25 (PE25) activated by c.3432 + 609 T > A, resulting in a frameshift and premature termination codon.

Two novel deep intronic SNVs were found to create cryptic splice sites. Analysis with the RNA Splicer tool predicted that these variants would induce aberrant splicing through pseudoexon inclusion. In Patient 14 (P14), the c.3432 + 609 T > A variant generated a cryptic donor site within intron 25. This donor site spliced to an ectopic acceptor site, leading to the insertion of a 134-bp pseudoexon (designated PE25) and resulting in a frameshift (Fig. 2). Immunofluorescence analysis confirmed the complete absence of dystrophin expression, as evidenced by the loss of signal for the rod domain (dystrophin-R), C-terminus (dystrophin-C), and N-terminus (dystrophin-N) (Fig. 4). Similarly, in Patient 28 (P28), the c.2804–5824 A>G variant activated a cryptic acceptor site in intron 21, causing the inclusion of pseudoexon PE21 and introducing a premature termination codon (PTC) (Supplementary Figure S2).

#### 3.2.2 Exon skipping

The c.358–13 T > G variant disrupted the canonical splice site. Prediction by the RNA Splicer tool indicated that this variant induces an aberrant splicing pattern characterized by exon skipping. In Patient 37 (P37), this variant weakened the acceptor site of exon 6, leading to the complete skipping of this exon. This event introduced a PTC (Supplementary Figure S3), a finding consistent with the absence of dystrophin signal observed in immunofluorescence analysis.

#### 3.2.3 Intron retention

The c.7873–11 A>G variant created a novel acceptor site within intron 53. The RNA Splicer tool predicted its effect as an aberrant splicing pattern characterized by intron retention. In Patient 18 (P18), RNA sequencing confirmed that this variant caused the retention of a 10-bp intronic segment, thereby introducing a PTC (Supplementary Figure S4).

### 3.3 Complex structural variations

#### 3.3.1 Inversions

**Fig. 3:**
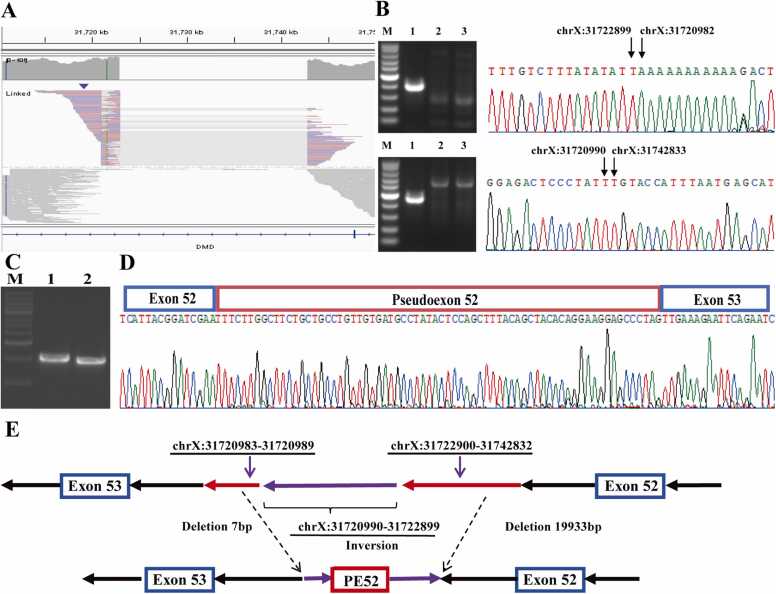
Characterization of the intronic inversion leading to pseudoexon inclusion in Patient 24 (P24). (A) Integrative Genomics Viewer (IGV) visualization of the inversion in P24. (B) Sanger sequencing validates the inversion (g.31720990_31722899inv) and associated deletions; PCR across breakpoints yields a product in P24 (lane 1) but not in controls (lanes 2–3). M, marker. (C) RT-PCR of exons 51–54 shows an aberrant fragment in P24 (lane 1) compared to the control (lane 2). M, 100 bp ladder. (D) Schematic of pseudoexon 52 (PE52) activated by the inversion, leading to frameshift and premature termination. (E) Graphical summary of the aberrant splicing induced by the intronic inversion.

**Fig. 4:**
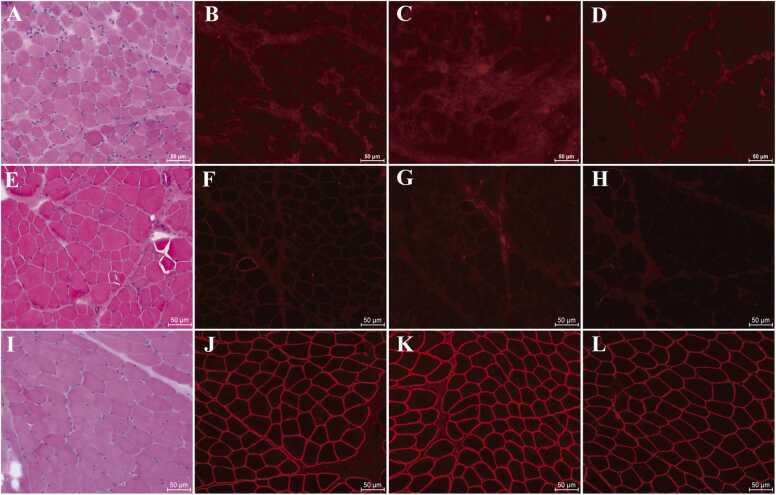
Histopathological and immunofluorescence features of skeletal muscle biopsies from P14 and P24. (A) H&E staining of P14 muscle tissue shows dystrophic morphology, including fiber size variation, endomysial fibrosis, and degenerating/regenerating fibers. (B–D) Immunofluorescence reveals complete loss of dystrophin-R (B), dystrophin-C (C), and dystrophin-N (D) in P14. (E) H&E staining of P24 muscle displays dystrophic changes with pronounced fiber size heterogeneity and degenerating/necrotic/regenerating fibers. (F–H) Immunofluorescence shows severe reduction of dystrophin-R (F) and complete absence of dystrophin-C (G) and dystrophin-N (H) in P24. (I) H&E staining of control muscle demonstrates normal fiber size uniformity. (J–L) Immunofluorescence confirms normal expression of dystrophin-R (J), dystrophin-C (K), and dystrophin-N (L) in the control.

Thirteen large inversions, ranging in size from 1.9 kb to 95 Mb, were resolved at nucleotide precision and validated by Sanger sequencing (Supplementary Figures S5–S14). A notable example was a 1.9 kb inversion within intron 52 of Patient 24 (P24), which activated a cryptic donor site adjacent to the native acceptor site, resulting in pseudoexon inclusion (Fig. 3). This 1.9 kb intronic inversion represents a previously unreported pathogenic mechanism in dystrophinopathies. Immunofluorescence analysis confirmed its functional impact, showing a severe reduction in dystrophin-R and a complete absence of dystrophin-C and dystrophin-N expression (Fig. 4).

#### 3.3.2 Translocations

We identified five balanced translocations that disrupted the genomic architecture of the *DMD* locus (Supplementary Figures S15–S19). In Patient 3 (P3), for instance, a translocation between *DMD* intron 2 (chrX:31,122,309) and TACC2 intron 5 (chr10:123,887,502) juxtaposed *DMD* exons 1–2 with regulatory elements from the TACC2 gene, thereby abolishing dystrophin expression (Supplementary Figure S17). All translocation breakpoints were confirmed by Sanger sequencing.

#### 3.3.3 Insertions/deletions

In Patient 23 (P23), a 99-kb insertion rich in LINE-1 repeats within intron 42 introduced a polyadenylation signal, leading to transcript truncation (Supplementary Figure S20). Conversely, a 30-bp deletion in Patient 30 (P30) within intron 25 exposed a cryptic acceptor site, resulting in the incorporation of a 48-bp pseudoexon (Supplementary Figure S21).

### 3.4 Carrier screening and segregation analysis

Sanger sequencing, based on the pathogenic *DMD* variants identified in the probands, confirmed carrier status in 27 of 32 mothers (84.4 %). One additional mother was classified as an obligate carrier, defined as having either at least two affected sons or pedigree-verified transmission of the variant (Supplementary Figure S1) [5], [15]. The overall maternal carrier rate was 84.8 % (28/33), which was significantly higher than the previously reported rate of 64.3 % [9] (two-tailed binomial test, *p* = 0.016).

Cascade testing among 27 maternal female relatives (excluding the probands' mothers) identified 10 molecularly confirmed carriers and 7 obligate carriers, yielding a combined carrier rate of 63.0 % (17/27). The carrier rates across different kinship categories were as follows: sisters (46.7 %, 7/15), maternal aunts (66.7 %, 2/3), grandmothers (83.3 %, 5/6), maternal cousins (100 %, 1/1), and great-grandmothers (100 %, 2/2) (Supplementary Figure S1).

## 4 Discussion

The expanding spectrum of deep intronic mutations in the *DMD* gene underscores the necessity of integrating advanced genomic and transcriptomic approaches to resolve the molecular complexity of dystrophinopathies. Consistent with recent studies, our findings demonstrate that deep intronic variants, particularly those inducing pseudoexon inclusion or disrupting splicing regulatory elements, represent a significant and previously underdiagnosed cause of dystrophinopathies. Such variants evade detection by conventional exon-focused methods such as MLPA and short-read exome sequencing, highlighting the importance of combining genomic LRS with muscle-derived RNA analysis to elucidate their molecular mechanisms and inform therapeutic strategies.

### 4.1 Technological advances in detecting structural and splice-disrupting variants

As shown in this and prior studies, genomic LRS enables precise identification of structural variants and complex rearrangements across the 2.2 Mb *DMD* locus [7], [14], [16]. For example, Okubo et al. reported chromosomal translocations and inversions disrupting the *DMD* gene, while Zhao et al. resolved two deep intronic deletions (c.6439–1016_6439–3376del and c.8669–19_8669–24del) using targeted LRS [14], [16]. In contrast to short-read sequencing, genomic LRS provides nucleotide-level resolution of breakpoint junctions and captures repetitive sequences such as LINE-1 insertions, which are known to contribute to hybrid pseudoexon formation. However, LRS alone cannot determine the transcriptional outcomes of such variants, underscoring the need for RNA-seq or RT-PCR to confirm aberrant splicing events. In this study, RNA-seq identified pseudoexon inclusions, exon skipping, and intron retention events that introduced premature termination codons (PTCs), findings that align with the observed loss of dystrophin expression in muscle biopsies. This combined DNA–RNA methodology supports recent diagnostic guidelines for resolving elusive dystrophinopathy mutations and reinforces the clinical value of parallel genomic and transcriptomic analysis [6], [11].

### 4.2 Mechanisms of aberrant splicing activation

Deep intronic variants contribute to aberrant splicing in dystrophinopathies through three primary mechanisms: (1) creation of de novo splice sites, (2) disruption of splicing enhancers or silencers, and (3) modification of branch points. In our cohort, 30 patients carried deep intronic SNVs that induced pseudoexon activation or exon skipping via the first two mechanisms, confirming the clinical relevance of these processes in dystrophinopathies. In contrast, branch point alterations appear to be rare in this disorder, as supported by our previous observations and reports from Xie et al. [14], [17]. The relatively low frequency of branch point mutations compared to other splicing defects may reflect evolutionary constraints on these regulatory elements, warranting further investigation into their sequence conservation and potential compensatory mechanisms.

This study also revealed two rare mechanisms leading to aberrant splicing. In Patient 23 (P23), a 99-kb insertion rich in LINE-1 repeats introduced a premature polyadenylation signal. This insertion may have induced transcriptional termination through LINE element-mediated interference, a phenomenon previously linked to retrotransposon-driven mutagenesis [6]. In Patient 24 (P24), an intronic inversion activated cryptic splice sites on the antisense strand, resulting in pseudoexon inclusion. These findings expand the known mutational spectrum of dystrophinopathies and underscore the underrecognized contribution of structural variation to splicing dysregulation.

Together, these discoveries broaden the genetic landscape of *DMD* mutations and highlight the importance of non-canonical splicing mechanisms in dystrophinopathy pathogenesis. Furthermore, they provide a mechanistic basis for developing therapeutic strategies targeting splicing defects.

### 4.3 Mapping mutation types to therapeutic modalities

The expanding molecular spectrum uncovered in this study highlights the need to align specific *DMD* mutation classes with appropriate therapeutic strategies. For canonical exonic deletions that disrupt the reading frame, antisense oligonucleotide (ASO)-mediated exon skipping represents the most clinically validated approach, capable of restoring a partially functional, Becker-like dystrophin isoform [18], [19]. However, this strategy is inherently mutation-specific and applicable only to subsets of deletions that can be converted to an in-frame transcript by skipping particular exons (e.g., 45, 51, or 53). Deep intronic variants that activate pseudoexons also represent promising targets for custom-designed ASOs. Such ASOs can be designed to block aberrant splice sites and restore normal splicing patterns. For instance, the c.3432 + 609 T > A, c.2804–5824 A>G, and c.3432 + 2050_3432 + 2079del variants identified in this study, each of which activates a cryptic splice site, could potentially be targeted by ASOs to prevent pseudoexon inclusion.

In contrast, AAV-mediated gene replacement offers a mutation-agnostic strategy that is theoretically applicable to all variant types, including point mutations, small insertions/deletions (indels), and complex structural variants [20]. For patients with complex structural variants, such as P12, who carried a large inversion spanning multiple exons, or P34, with an interchromosomal translocation, gene replacement represents a necessary therapeutic avenue. However, this approach faces technical challenges, primarily the ≈ 4.7 kb packaging limit of conventional AAV vectors and the high prevalence of neutralizing antibodies that restrict both initial and repeated dosing. To overcome these limitations, dual- and triple-AAV systems have been developed to deliver split dystrophin transgenes, enabling partial or near-complete restoration of dystrophin expression *in vivo* [20]. More recently, refined split-intein-mediated protein trans-splicing strategies have further enhanced reconstitution efficiency [21], [22]. Building on these multi-vector systems, next-generation single-vector AAV platforms with expanded cargo capacity are under exploration to transcend the inherent packaging constraints of conventional vectors.

Beyond gene replacement, CRISPR/Cas-based genome editing holds transformative potential for achieving permanent correction at the genomic level [23], [24]. For single-nucleotide variants, such as several novel SNVs identified in this study (Table S2), base editing or prime editing systems can precisely revert pathogenic nucleotides without inducing double-strand DNA breaks. Nevertheless, the safe and efficient delivery of these large editing machineries to skeletal and cardiac muscle remains a substantial challenge. Ongoing advances in large-capacity AAV systems, muscle-tropic capsid engineering, and exosome-mediated mRNA delivery are actively addressing existing limitations in packaging capacity and immunogenicity.

Together, these therapeutic innovations signify a paradigm shift from descriptive molecular diagnosis toward actionable precision medicine. In this emerging framework, genotype-specific insights can rationally guide the selection and application of ASO, AAV, or CRISPR-based platforms to enable personalized treatment of dystrophinopathies.

### 4.4 Screening of female relatives for DMD carrier status

Duchenne muscular dystrophy (DMD), while rare (affecting approximately 1 in 3600–6000 male births), imposes a profound socioeconomic burden. In high-income countries such as Germany, Italy, the United Kingdom, and the United States, the total annual societal cost per patient has been estimated between $80,120 and $120,910, increasing substantially with disease progression, while the corresponding household financial burden ranges from $58,440 to $71,900 [25]. This considerable economic impact, combined with the life-threatening nature of the disease and the requirement for lifelong multidisciplinary care, highlights the importance of systematic carrier screening among female relatives of affected individuals. Such screening forms a critical component of preventive strategies aimed at optimizing resource allocation and providing targeted support to affected families [3], [26].

Conventional diagnostic approaches, including MLPA and ES, fail to detect approximately 2–5 % of pathogenic *DMD* variants due to technical limitations in resolving complex structural variants and deep intronic single nucleotide variants. A representative example is Family 12 (F12): although the proband (F12-Ⅲ1) was diagnosed by muscle biopsy before the birth of F12-Ⅲ7, both MLPA and ES failed to identify the underlying *DMD* variant. As a result, carrier testing was not available to the family, which subsequently led to the birth of a second affected child (F12-Ⅲ7).

LRS addresses these diagnostic limitations by enabling comprehensive detection of complex structural variants and single nucleotide variants across the 2.2-Mb *DMD* locus. In this study, LRS-guided carrier screening identified 45 carriers or obligate carriers, who were subsequently stratified into distinct management pathways [27], [28], [29], [30]: (1) Reproductive-age carriers received immediate interventions, such as preimplantation genetic testing for monogenic disorders (PGT-M) during in vitro fertilization (IVF) or prenatal diagnosis for ongoing pregnancies. (2) Pre-reproductive carriers were enrolled in longitudinal counseling programs to support future reproductive planning. (3) Post-reproductive carriers (e.g., grandmothers and great-grandmothers) facilitated cascade screening of at-risk relatives, thereby helping to prevent further variant transmission. These results support the integration of LRS into routine clinical workflows to overcome the inherent limitations of conventional MLPA and ES. Given its potential to prevent variant transmission through proactive family testing, all female relatives in multigenerational pedigrees, irrespective of their current reproductive plans, should be prioritized for genetic counseling and, where appropriate, molecular screening.

### 4.5 Limitations and future directions

While the integration of LRS and RNA-seq has demonstrated considerable effectiveness in identifying genomic variants and their transcriptional consequences in dystrophinopathy patients, several limitations remain. First, LRS requires genomic DNA of high integrity, a more stringent requirement than that of conventional methods such as MLPA or short-read sequencing. Moreover, the analysis of long-read data relies on specialized bioinformatics tools for sequence alignment, isoform detection, and structural variant calling, presenting technical and operational challenges for widespread clinical adoption. Additionally, RNA-seq is contingent upon the availability of high-quality RNA, which is often difficult to obtain in practice due to the invasive nature of muscle biopsies. As noted by Pan et al., *DMD* expression in whole blood is negligible, making muscle tissue the currently necessary source for reliable transcriptomic analysis [31]. The integrity of RNA can be further compromised by suboptimal preservation or sample degradation, which may in turn reduce diagnostic accuracy.

Beyond technical challenges, the interpretation of putative hotspot variants such as c.9225–647 A>G is constrained by sample size and population diversity. Its recurrence in a small, ethnically homogeneous Chinese cohort (n = 50) limits statistical power and raises the possibility of sampling bias. Validation in larger, multi-ethnic populations is therefore needed to confirm its biological and clinical relevance.

Advances in sequencing technologies and computational methods are expected to help address these limitations. Improvements in the cost-effectiveness and accuracy of LRS could broaden its accessibility, while enhanced bioinformatics tools may simplify structural variant detection and isoform quantification. The integration of multi-omics data, such as combining RNA-seq with proteomic or epigenetic profiling, could further improve the functional interpretation of non-coding variants and support more precise therapeutic targeting. In parallel, efforts to identify less invasive alternatives to muscle biopsies may alleviate current sampling constraints. Cultured skin fibroblasts, for example, represent a promising surrogate tissue for *DMD* transcriptomic analysis, as they express measurable levels of *DMD* transcripts and have been shown to recapitulate splicing abnormalities observed in muscle [32]. Exon trapping assays also offer a functional approach to assess the impact of variants on *DMD* pre-mRNA splicing. Future large-scale, multi-ethnic, and longitudinal studies integrating these innovations will be essential to refine genotype–phenotype correlations, validate novel pathogenic mechanisms, and accelerate the development of precision therapies for dystrophinopathies.

## 5 Conclusion

This study underscores the pathogenic significance of deep intronic variants in dystrophinopathies and demonstrates the value of an integrated diagnostic approach combining long-read sequencing, RNA sequencing, and advanced bioinformatics. By delineating specific mechanisms of pseudoexon activation and transcriptional disruption, our findings provide important insights into the non-canonical splicing landscape of the *DMD* gene. Together, these advances establish a foundation for developing precision therapies that target aberrant splicing events and strengthen the translational bridge connecting genomic discovery to clinical application.

## Ethical approval statement

This study was approved by the Research Ethics Board of Children's Hospital of Fudan University (Approval No. [2022]300 A). All participants (or parent/legal guardians) provided written informed consent. Additionally, all relatives undergoing cascade testing provided voluntary written informed consent after being fully informed of the purpose, potential risks, limitations and implications of genetic testing. Declinations to participate were respected and documented. For minors, both parental/guardian permission and the minor's assent (where developmentally appropriate) were required. Post-test genetic counseling was provided to all participants, and identified carriers were referred for appropriate clinical follow-up.

## Funding information

This work was supported by the National Key R&D Program of China [No. 2022YFC2703600].

## CRediT authorship contribution statement

**Lei Zhao:** Conceptualization, Data curation, Investigation, Methodology, Resources, Validation, Visualization, Writing – original draft, Writing – review & editing. **Shirang Pan:** Conceptualization, Investigation, Methodology, Validation, Visualization, Writing – original draft, Writing – review & editing. **Chaoping Hu:** Data curation, Resources, Validation. **Yiyun Shi:** Data curation, Resources, Validation. **Xihua Li:** Conceptualization, Data curation, Funding acquisition, Project administration, Supervision, Validation, Writing – review & editing.

## Declaration of Competing Interest

All authors declare that they have no conflicts of interest.

## Data Availability

The datasets generated and analyzed during this study are available from the corresponding author upon reasonable request. Data are stored in a controlled-access repository at the Children's Hospital of Fudan University. A subset of clinical data that does not compromise participant privacy is included in the manuscript or supplementary files. Researchers interested in accessing the full dataset may contact the corresponding author with a detailed research proposal outlining the intended use. All variant data were submitted to the Leiden Open Variation Database (LOVD) at: https://databases.lovd.nl/shared/genes/DMD.
