# Supplementary material for: From shadows to clarity: A new paradigm for comprehensive variant detection in undiagnosed dystrophinopathy using combined long-read and RNA sequencing
Source: Comput Struct Biotechnol J. 2025 Oct 24;27:5036–44. doi: 10.1016/j.csbj.2025.10.049 (PMC12663668; doi:10.1016/j.csbj.2025.10.049)
Supplement: Supplementary Table S1 — Supplementary material [file mmc1.pdf]

Supplementary Figures

Fig. S1 Pedigrees of fifty unrelated families with dystrophinopathies.

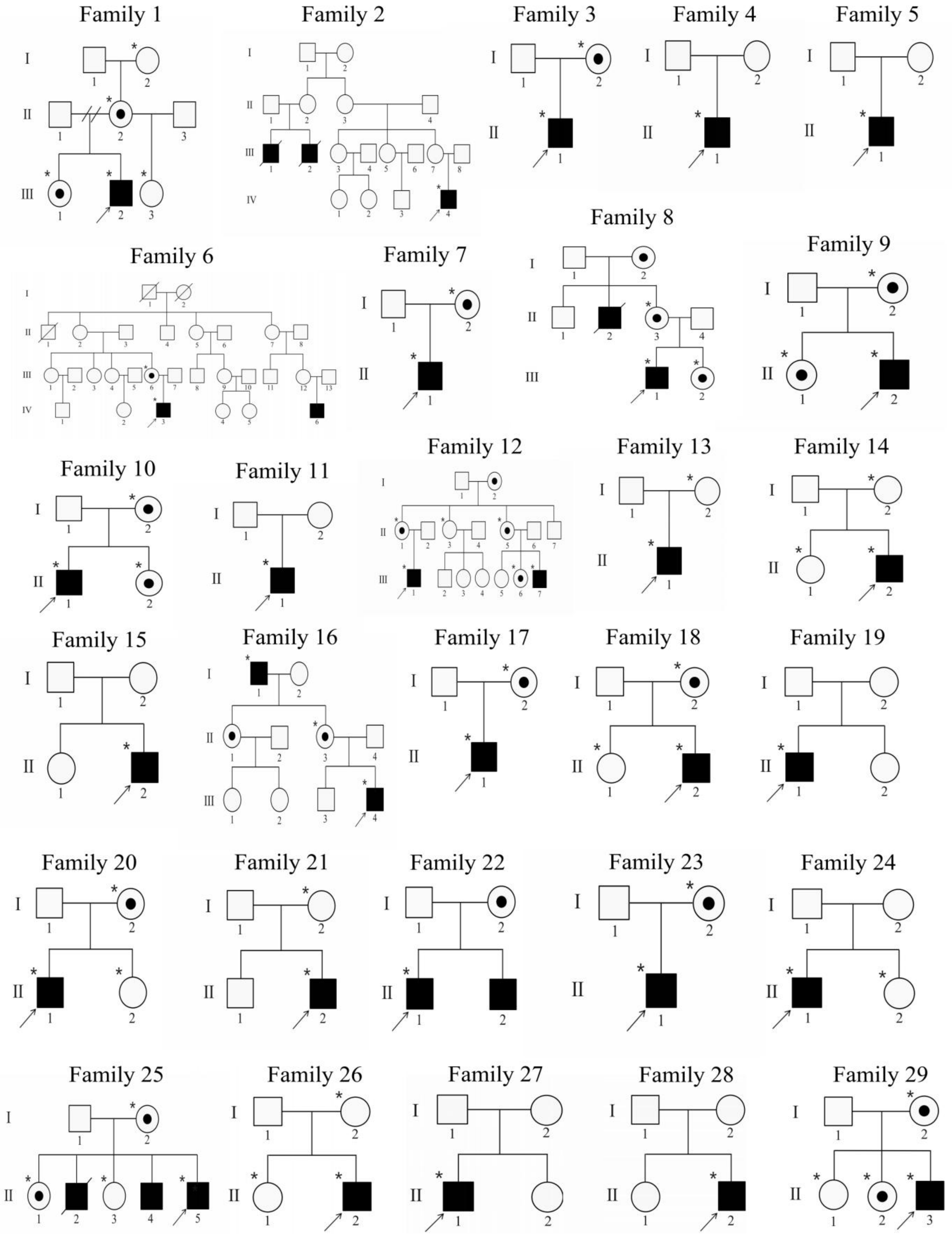

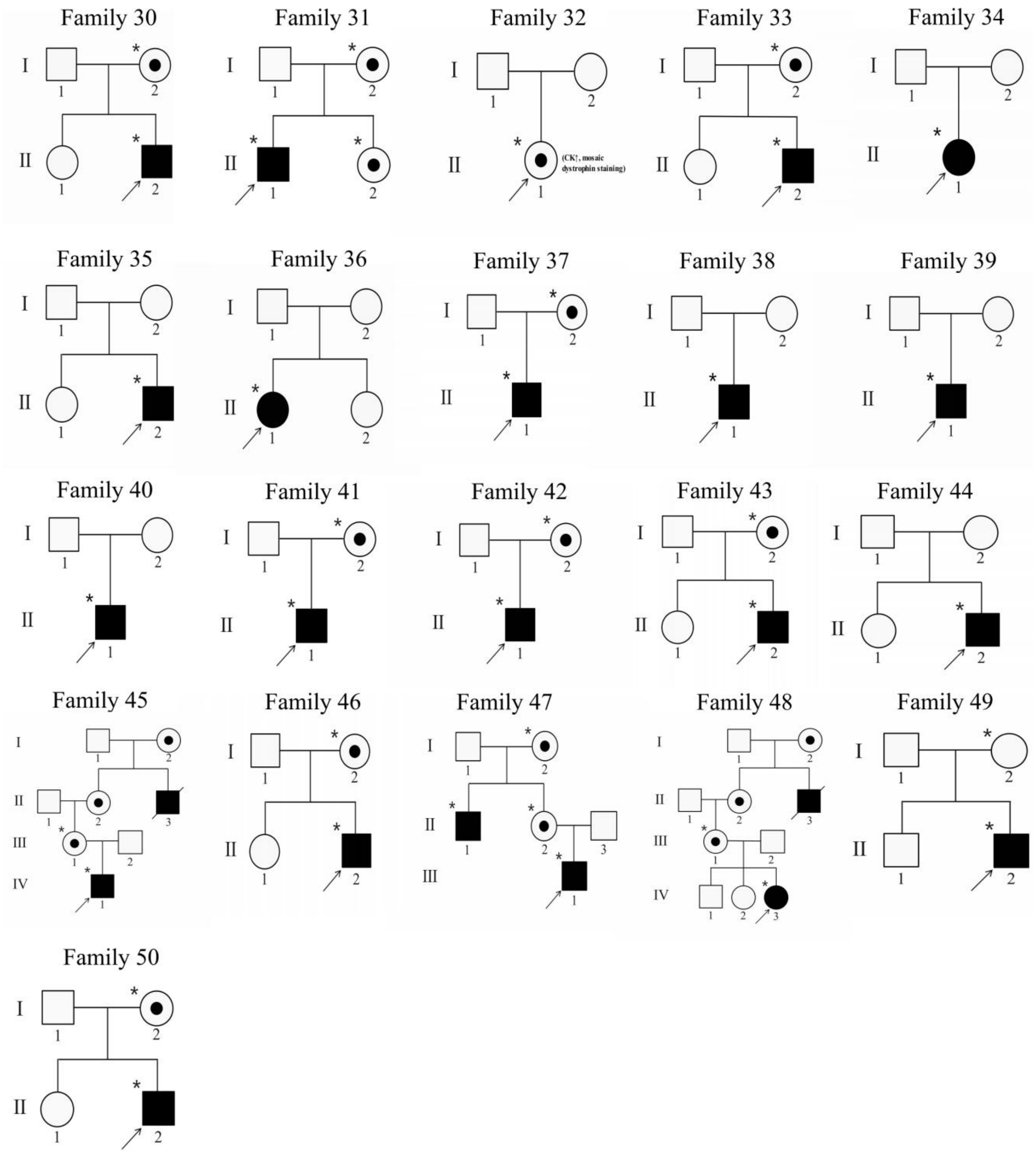

Affected individuals are denoted by black shading, with arrows indicating the index cases. Tested individuals are marked with an asterisk, while heterozygous female carriers are represented by a black dot within the female symbol.

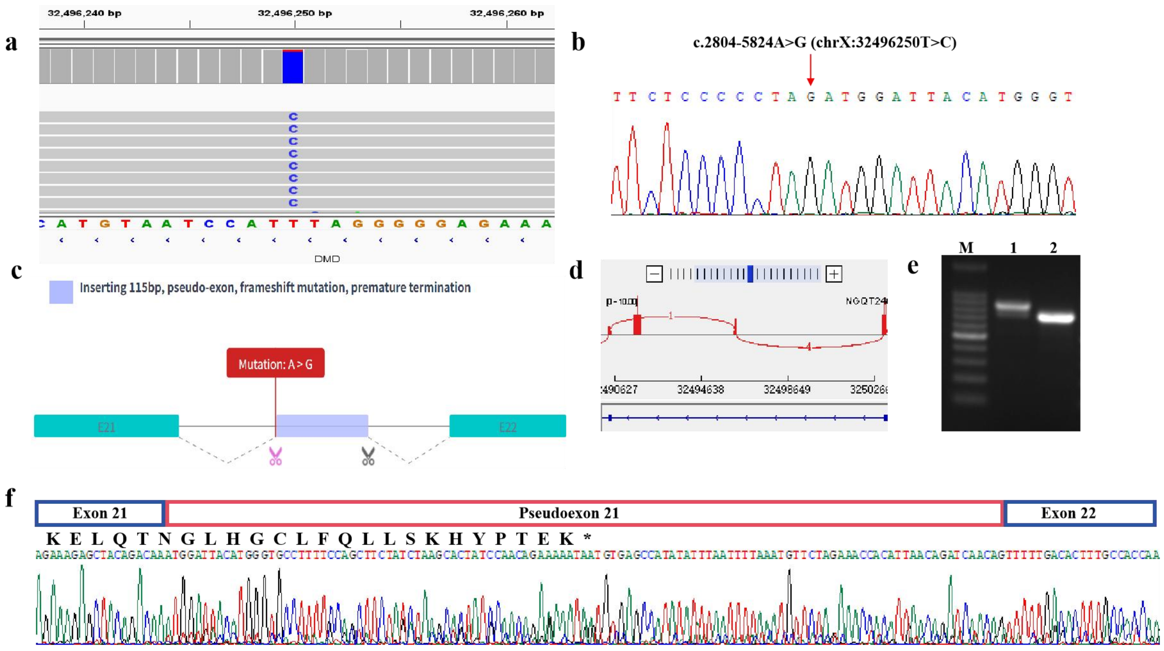

Fig. S2 Characterization of variant c.2804-5824A>G causing pseudoexon inclusion.

(a) Long-read sequencing of the genomic DNA of P28 showed a single-nucleotide variant c.2804-5824A>G (chrX:32496250T>C). **(b)** Sanger sequencing of the genomic DMD sequence of P28 confirmed the single-nucleotide variant c.2804-5824A>G (chrX:32496250T>C). **(c)** The online RNA Splicer tool predicted an aberrant splicing pattern caused by the deep intronic variant of c.2804-5824A>G. **(d)** Sashimi plot visualization showed that a pseudoexon was caused by the variant. **(e)** RT-PCR products spanning exons 20 – 23 showed an abnormal-sized fragment in P28 (lane 1), absent in a healthy control (lanes 2). M, size marker (100 bp ladder). **(f)** Schematic of pseudoexon 21 activated by the c.2804-5824A>G variant. The activated pseudoexon 21 causes frameshift and premature termination.

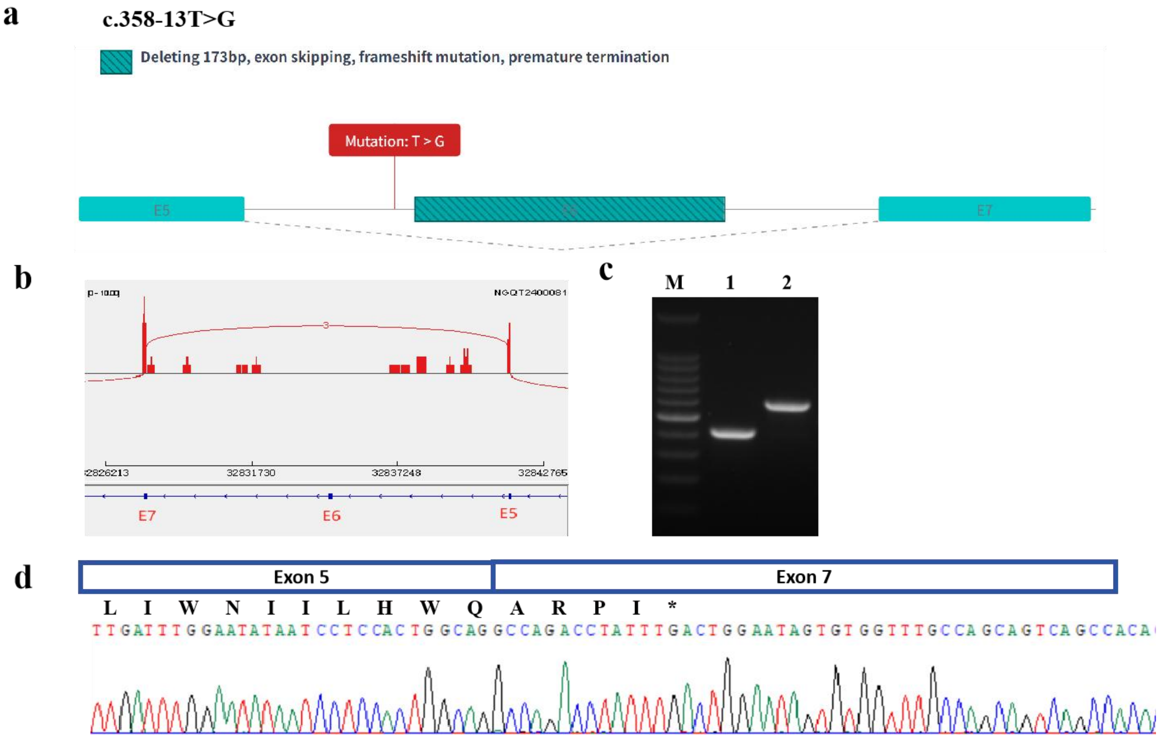

Fig. S3 Characterization of variant c.358-13T>G causing exon skipping.

(a) The online RNA Splicer tool predicted an aberrant splicing pattern caused by the intronic variant of c.358-13T>G. **(b)** Sashimi plot visualization of the RNA sequencing data showed that exon 6 skipping occurred in P37. **(c)** RT-PCR products spanning exons 4 – 6 showed an abnormal-sized fragment in P37 (lane 1), absent in a healthy control (lane 2). M, size marker (100 bp ladder). **(d)** Schematic of the exon 6 skipping activated by the c.358-13T>G variant.The exon 6 skipping causes frameshift and premature termination.

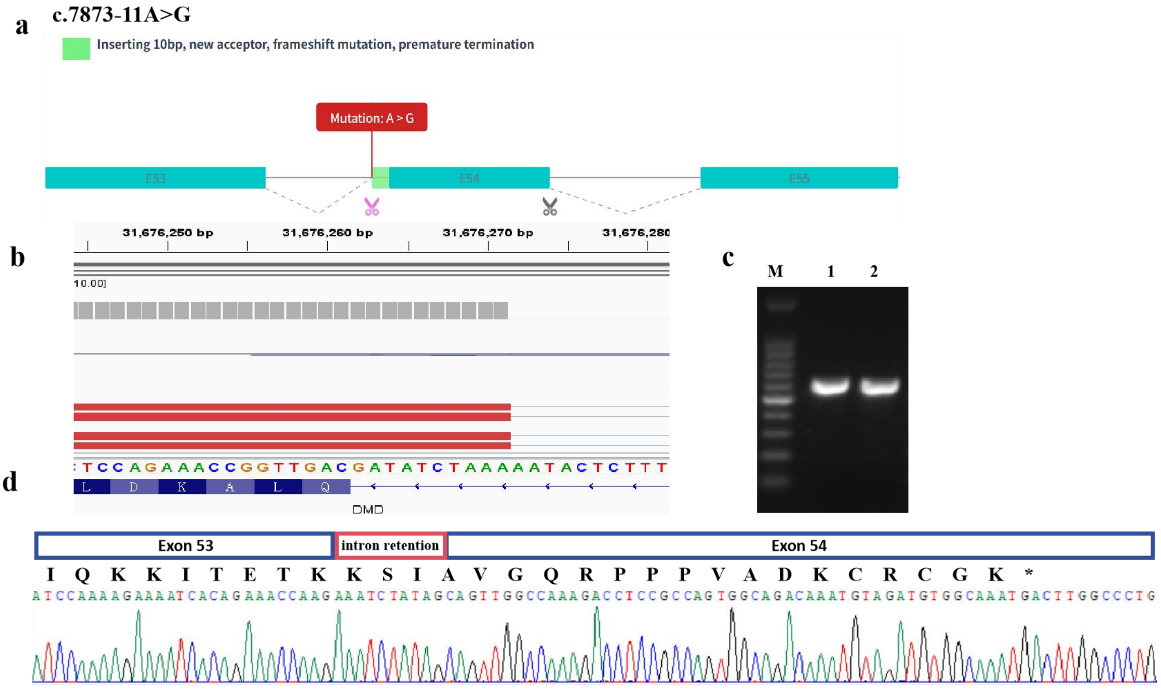

Fig. S4 Characterization of variant c.7873-11A>G causing partial intron retention.

(a) The online RNA Splicer tool predicted an aberrant splicing pattern caused by the intronic variant of c.7873-11A>G. **(b)** RNA sequencing showed retention of part of the intron in the transcript for P18. **(c)** RT-PCR products spanning exons 52–55 in P18 (lane 1) and a healthy control (lane 2). M, size marker (100 bp ladder). **(d)** Schematic of the partial intron retention activated by the c.7873-11A>G variant. The partial intron retention causes frameshift and premature termination.

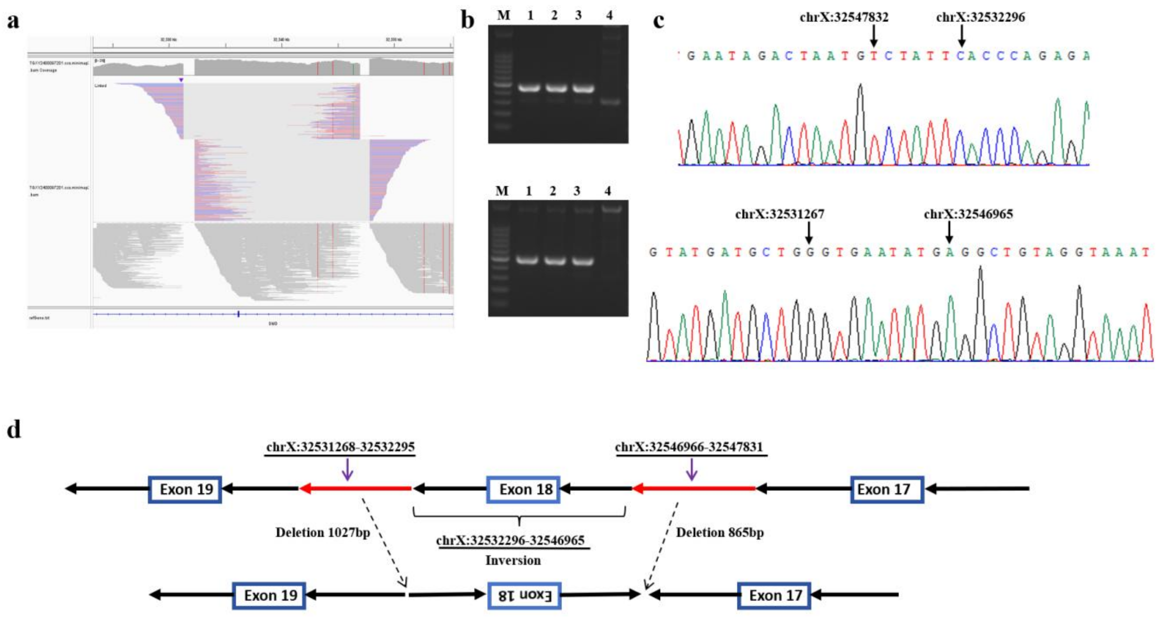

Fig. S5 Characterization of the inversion variant in P8.

(a) Long-read sequencing of the genomic DNA of P8 showed an inversion variant (g.32532296_32546965inv; g.32531268_32532295del; g.32546966_32547831del). **(b)** PCR products spanning the inversion breakpoints showed an expected fragment in P8, his mother, and his sister (lanes 1 – 3), absent in a healthy control (lanes 4). M, size marker (100 bp ladder). **(c)** Sanger sequencing of the PCR products confirmed the inversion variant. **(d)** Graphical representation of the inversion variant.

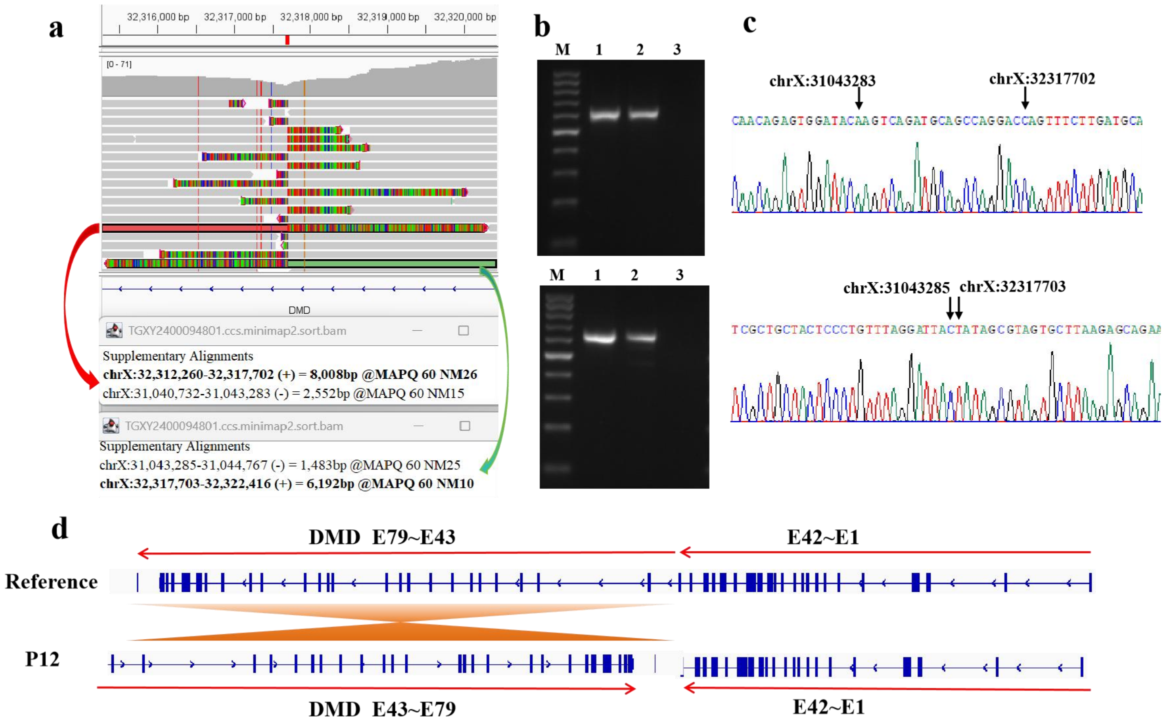

Fig. S6 Characterization of the inversion variant in P12.

(a) Long-read sequencing of the genomic DNA of P12 showed an inversion variant (g.31043285_32317703inv). **(b)** PCR products spanning the inversion breakpoints showed an expected fragment in P12 and his mother (lanes 1–2), absent in a healthy control (lanes 3). M, size marker. **(c)** Sanger sequencing of the PCR products confirmed the inversion variant. **(d)** Graphical representation of the inversion variant.

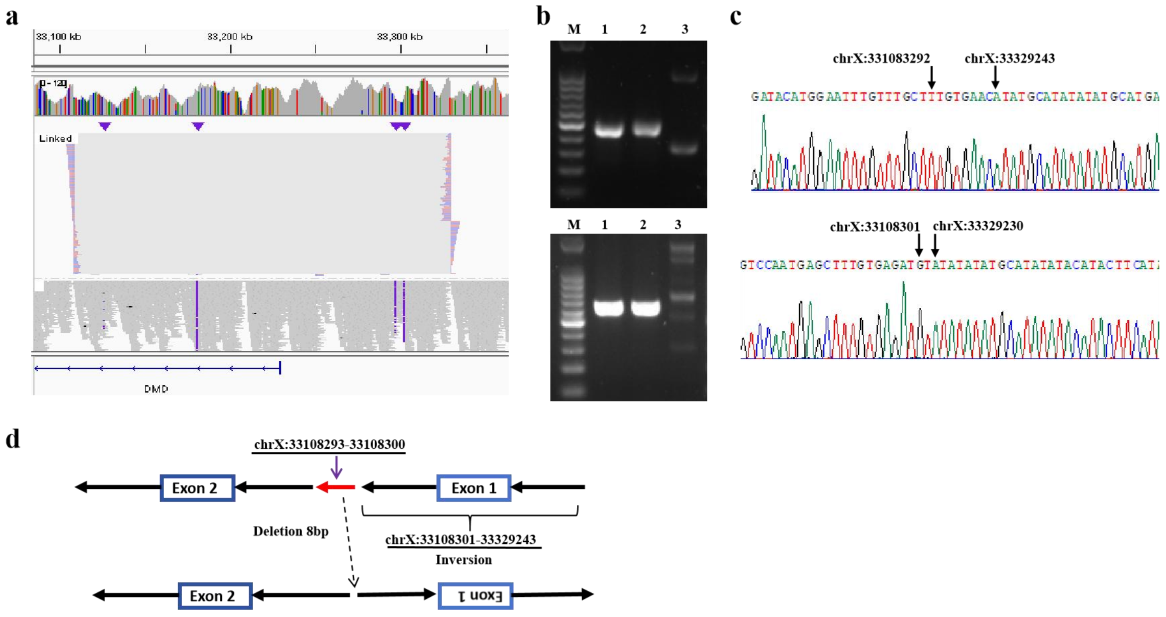

Fig. S7 Characterization of the inversion variant in P17.

(a) Long-read sequencing of the genomic DNA of P17 showed an inversion variant (g.33108301_33329243inv; g.33108293_33108300del). **(b)** PCR products spanning the inversion breakpoints showed an expected fragment in P17 and his mother (lanes 1 – 2), absent in a healthy control (lanes 3). M, size marker. **(c)** Sanger sequencing of the PCR products confirmed the inversion variant. **(d)** Graphical representation of the inversion variant.

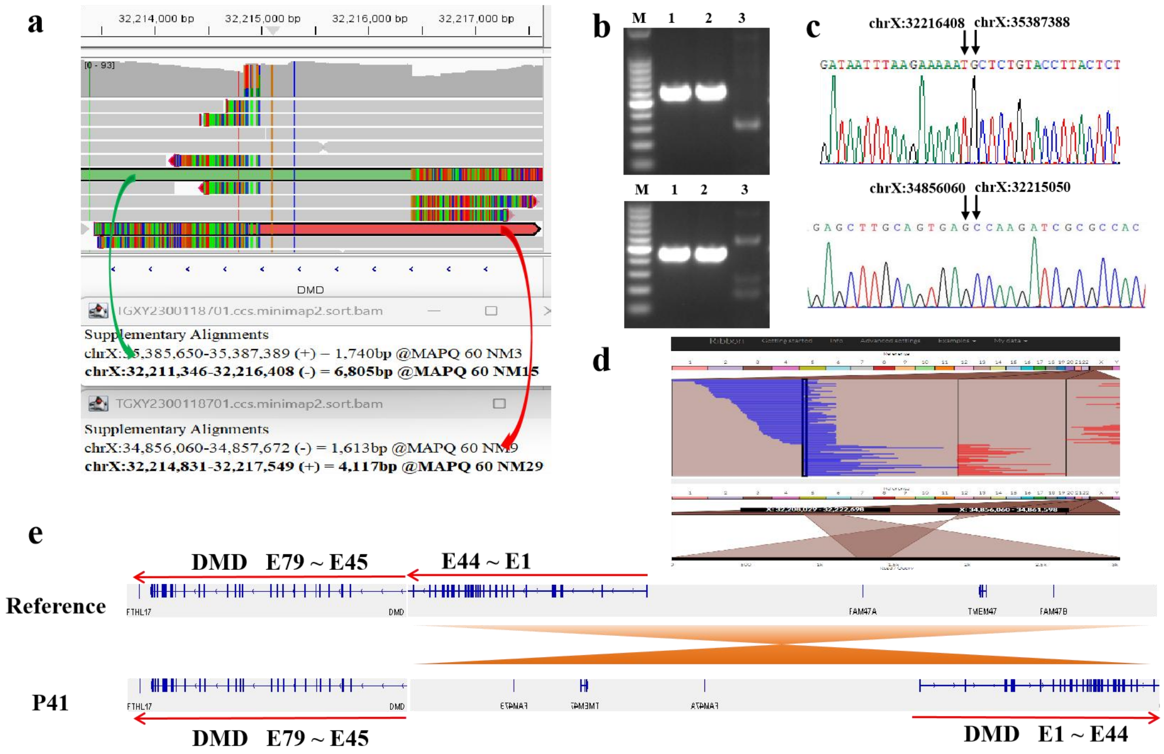

Fig. S8 Characterization of the inversion variant in P41.

(a) Long-read sequencing of the genomic DNA of P41 showed an inversion variant (g.32216408_35387388inv). **(b)** PCR products spanning the inversion breakpoints showed an expected fragment in P41 and his mother (lanes 1 – 2), absent in a healthy control (lanes 3). M, size marker. **(c)** Sanger sequencing of the PCR products confirmed the inversion variant. **(d)** Ribbon illustration of the inversion variant. **(e)** Graphical representation of the inversion variant.

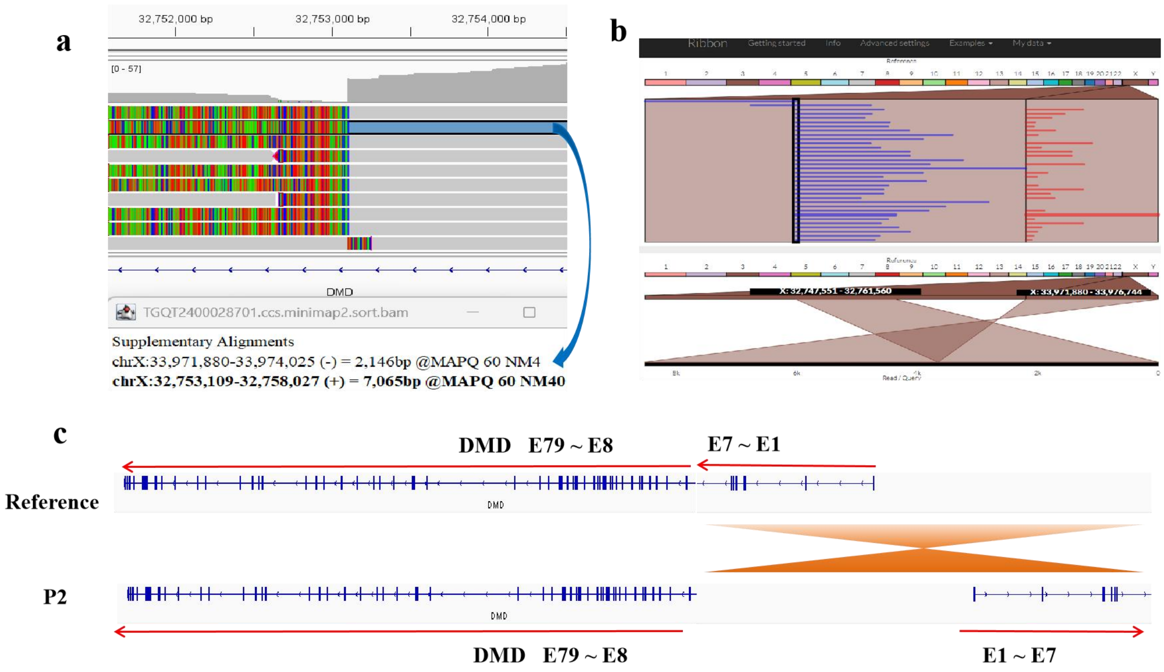

Fig. S9 Characterization of the inversion variant in P2.

(a) Long-read sequencing of the genomic DNA of P2 showed an inversion variant (g.32753109_33971880inv). **(b)** Ribbon illustration of the inversion variant. **(c)** Graphical representation of the inversion variant.

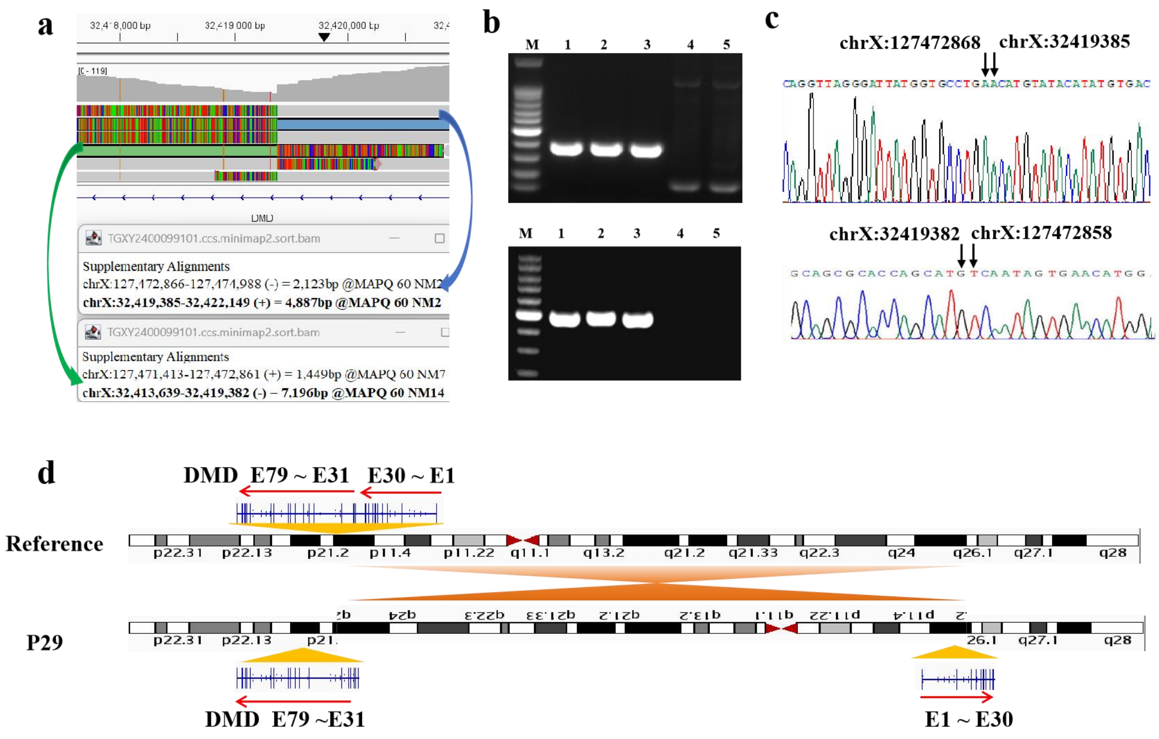

Fig. S10 Characterization of the inversion variant in P29.

(a) Long-read sequencing of the genomic DNA of P29 showed an inversion variant (g.32419385_127472868inv; g.32419383_32419384del). **(b)** PCR products spanning the inversion breakpoints showed an expected fragment in P29, his mother, and his sister (lanes 1-3), absent in a healthy control (lanes 4-5). M, size marker. **(c)** Sanger sequencing of the PCR products confirmed the inversion variant. **(d)** Graphical representation of the inversion variant.

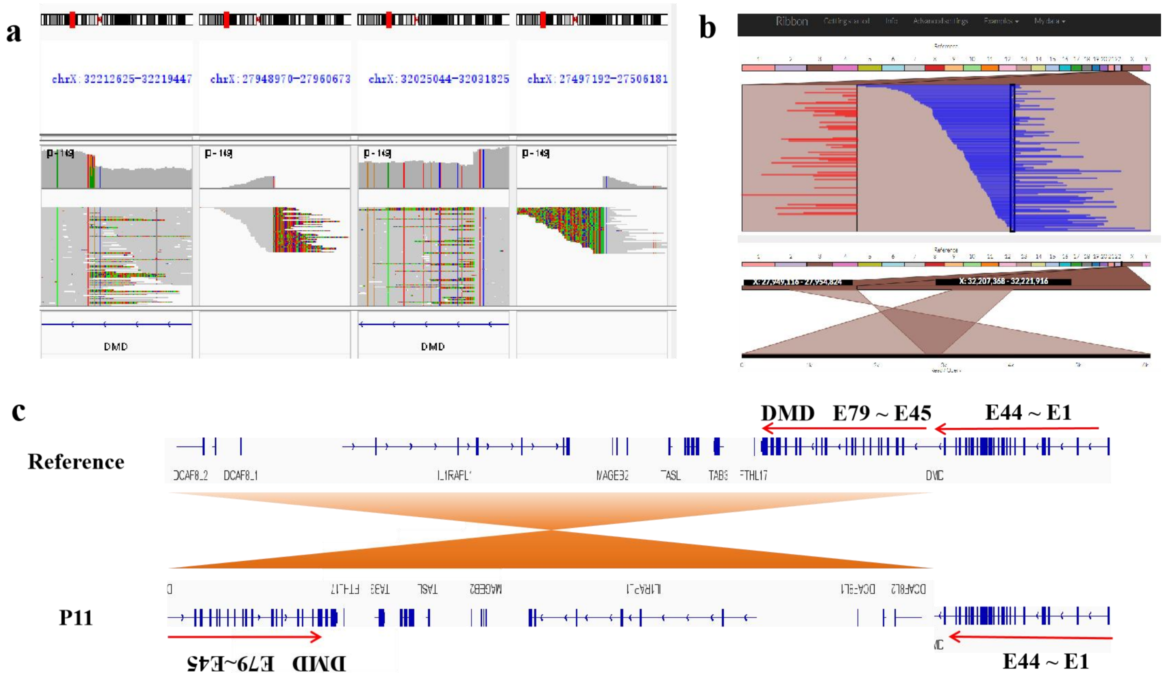

Fig. S11 Characterization of the inversion variant in P11.

(a) Long-read sequencing of the genomic DNA of P11 showed an inversion variant (g.27502401_32215080inv; g.32030296_32215080dup). **(b)** Ribbon illustration of the inversion variant. **(c)** Graphical representation of the inversion variant.

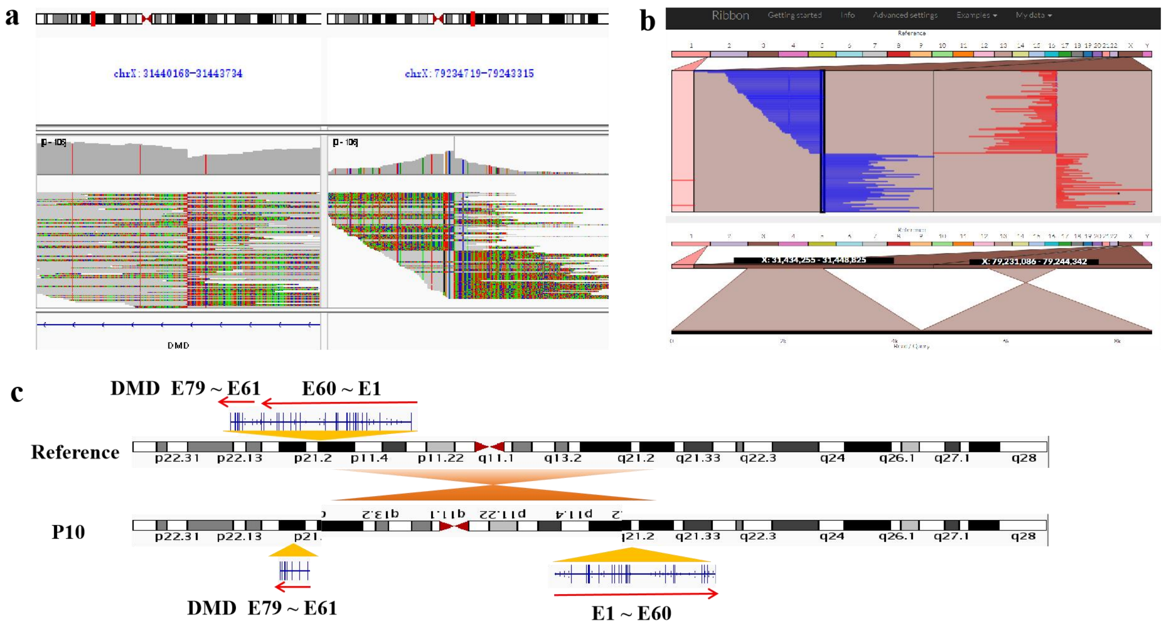

Fig. S12 Characterization of the inversion variant in P10.

(a) Long-read sequencing of the genomic DNA of P10 showed an inversion variant (g.31442071_79238585inv; g.31442070del). **(b)** Ribbon illustration of the inversion variant. **(c)** Graphical representation of the inversion variant.

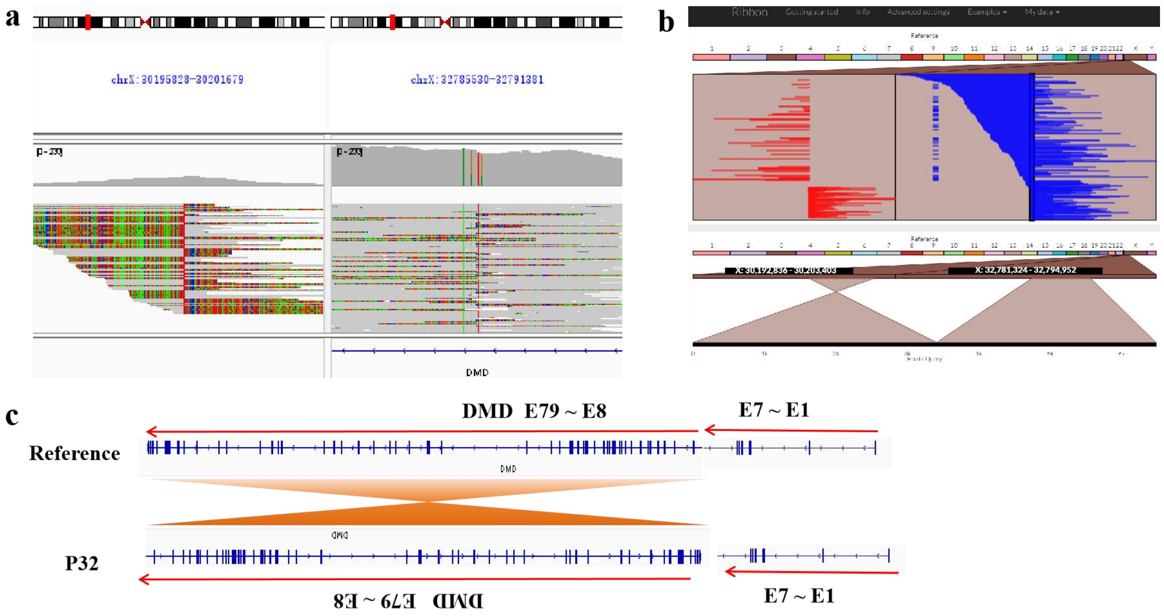

Fig. S13 Characterization of the inversion variant in P32.

(a) Long-read sequencing of the genomic DNA of P32 showed an inversion variant (g.30198889_32788457inv). **(b)** Ribbon illustration of the inversion variant. **(c)** Graphical representation of the inversion variant.

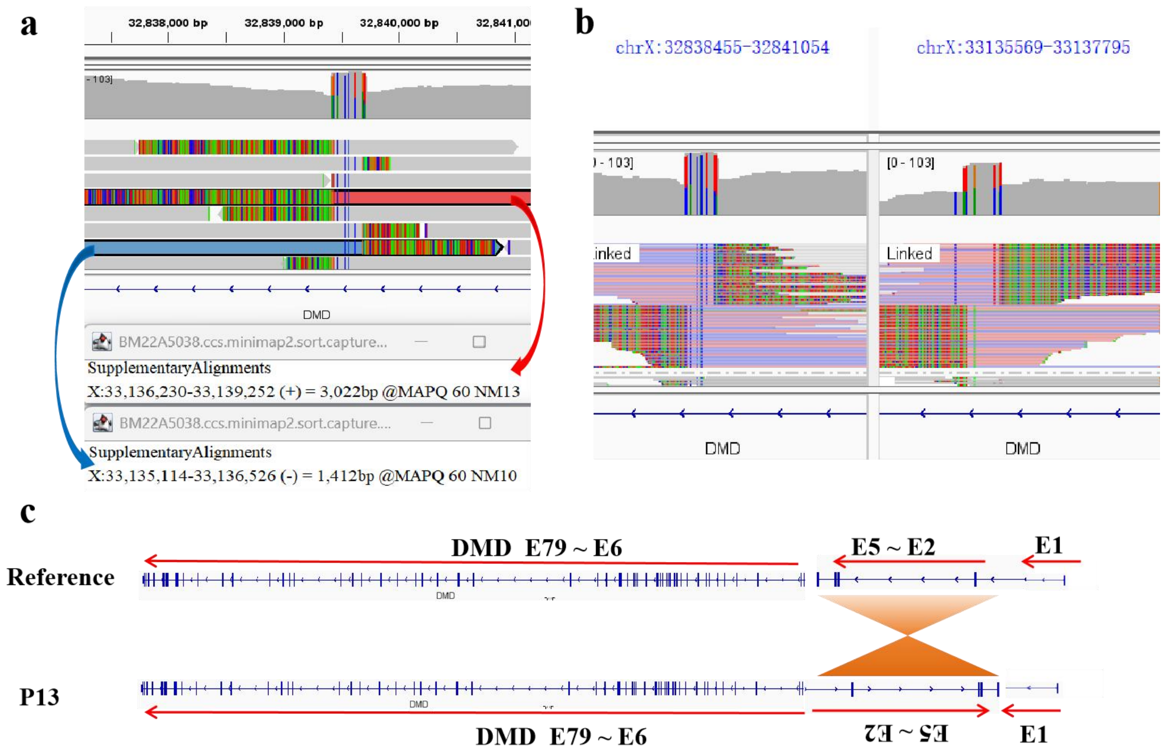

Fig. S14 Characterization of the inversion variant in P13.

(a, b) Long-read sequencing of the genomic DNA of P13 showed an inversion variant (g.32839627_33136469inv). **(c)** Graphical representation of the inversion variant.

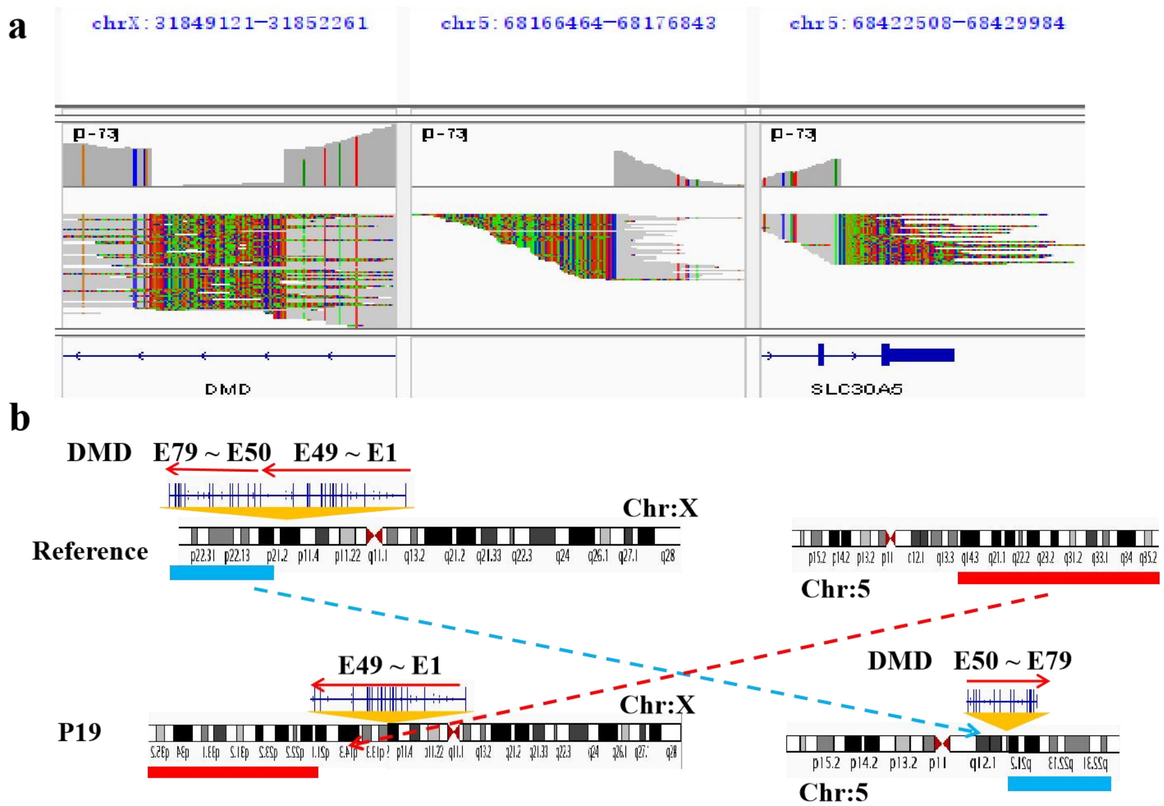

Fig. S15 Characterization of the translocation variant in P19.

(a) Long-read sequencing of the genomic DNA of P19 showed a translocation variant (NC_000023.10:g.pter_31849970delins[NC_000005.9:g.qter_68172884]; NC_000023.10:g.pter_31851240delins[NC_000005.9:g.qter_68424329]).

(b) Graphical representation of the translocation variant.

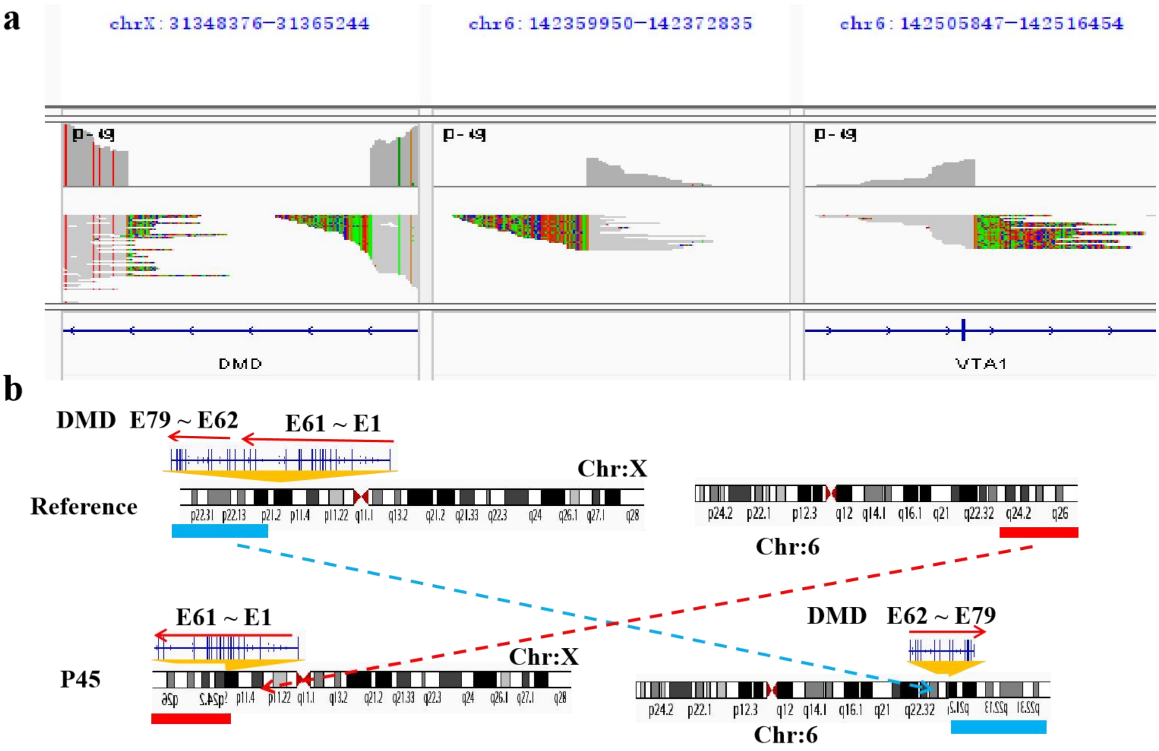

Fig. S16 Characterization of the translocation variant in P45.

(a) Long-read sequencing of the genomic DNA of P45 showed a translocation variant (NC_000023.10:g.pter_31351652delins[NC_000006.11:g.qter_142510955]; NC_000023.10:g.pter_31363178delins[NC_000006.11:g.qter_142365579]).

(b) Graphical representation of the translocation variant.

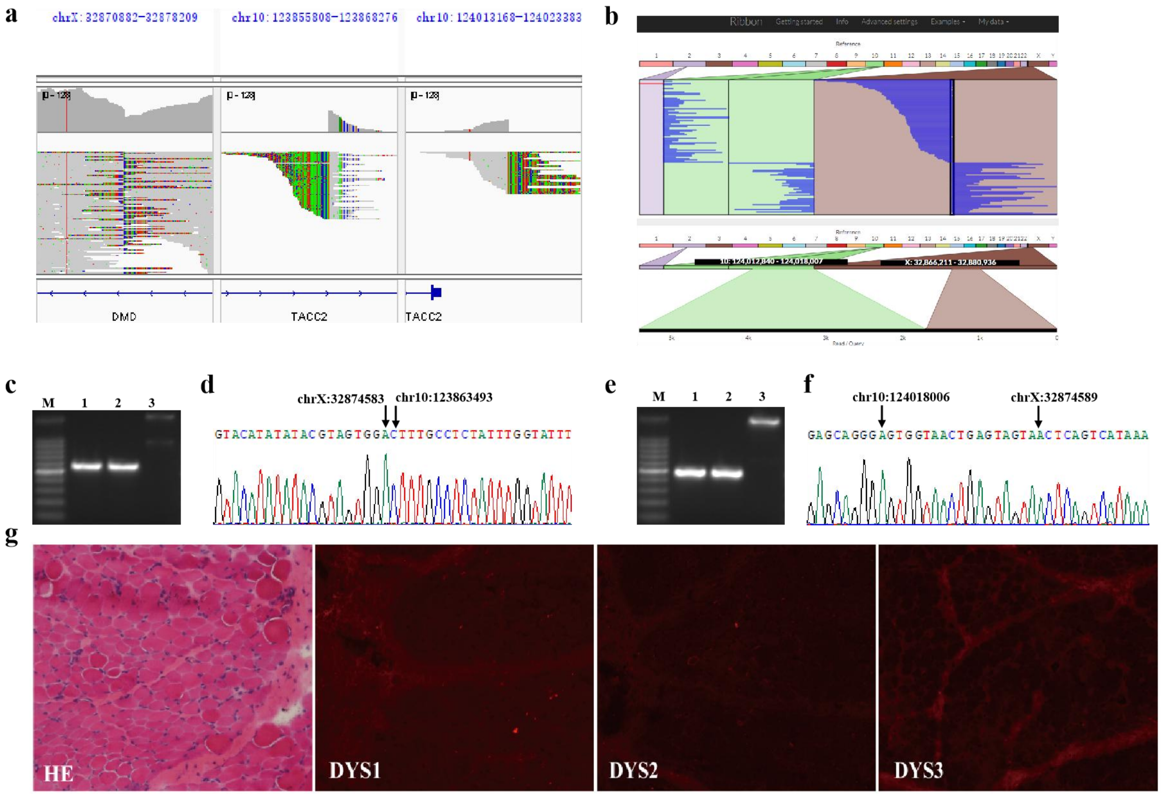

Fig. S17 Characterization of the translocation variant in P3.

(a) Long-read sequencing of the genomic DNA of P3 showed a translocation variant (NC_000023.10:g.pter_32874583delins[NC_000010.10:g.qter_123863493]; NC_000023.10:g.pter_32874589delins[NC_000010.10:g.qter_124018006]).

(b) Ribbon illustration of the translocation variant. **(c, e)** PCR products spanning the translocation breakpoints showed an expected fragment in P3 and his mother (lanes 1 – 2), absent in a healthy control (lanes 3). M, size marker. **(d, f)** Sanger sequencing of the PCR products confirmed the translocation variant. **(g)** H&E staining of muscle tissue shows dystrophic morphology, including fiber size variation, endomysial fibrosis, and degenerating/regenerating fibers. Immunofluorescence (DYS1, DYS2, DYS3) confirmed that dystrophin expression was absent.

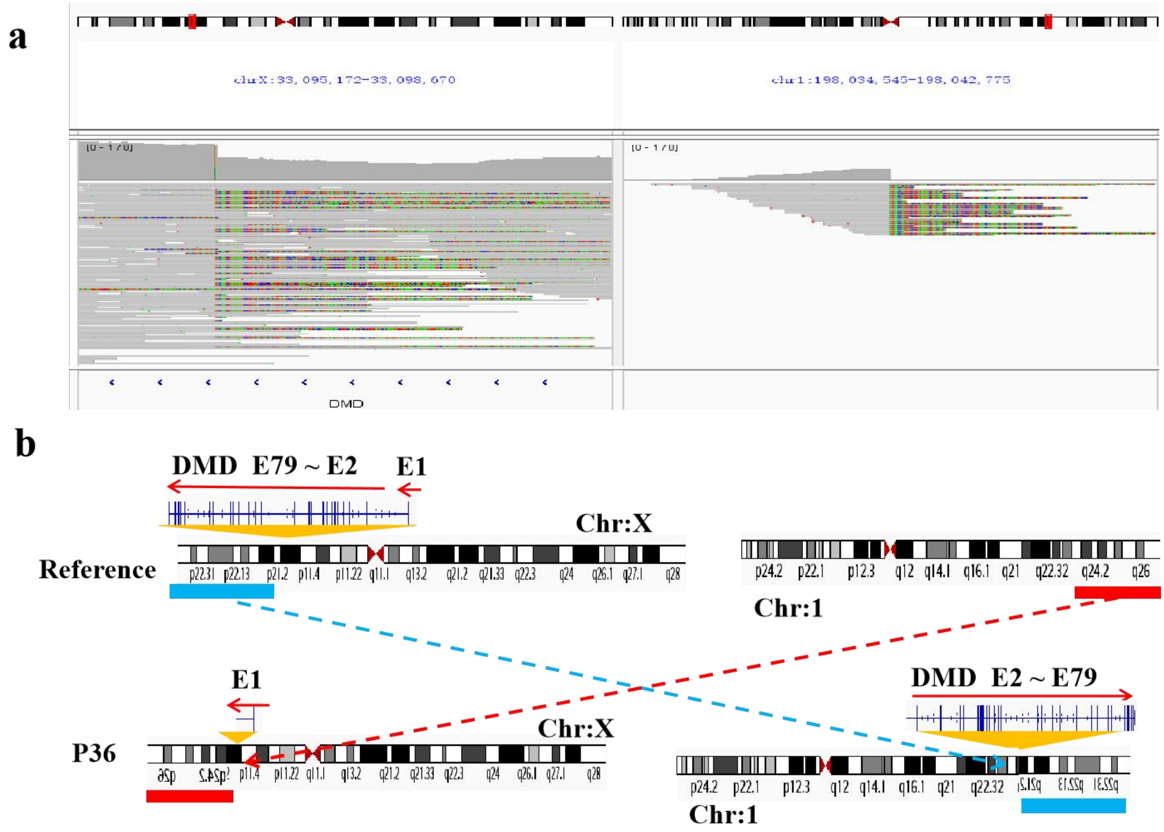

Fig. S18 Characterization of the translocation variant in P36.

(a) Long-read sequencing of the genomic DNA of P36 showed a translocation variant NC_000023.10:g.pter_33096082delins[NC_000001.10:g.qter_198038663]

(b) Graphical representation of the translocation variant.

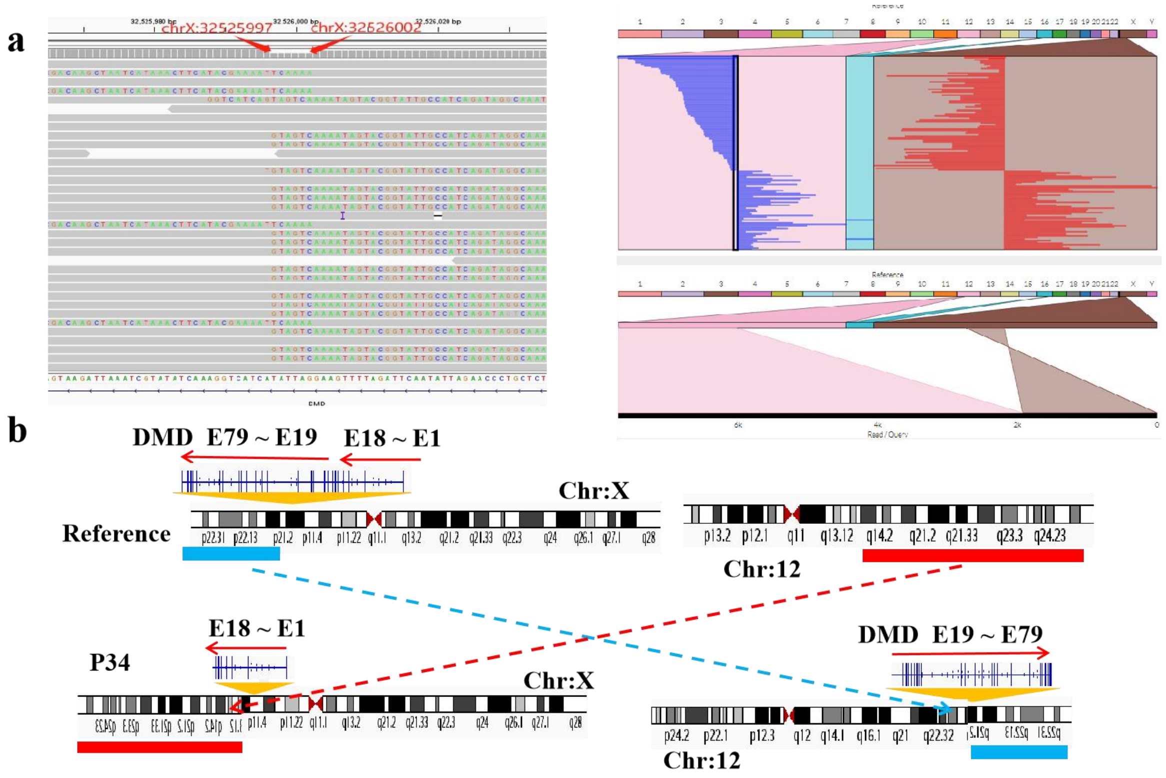

Fig. S19 Characterization of the translocation variant in P34.

(a) Long-read sequencing of the genomic DNA of P34 showed a translocation variant NC_000023.10:g.pter_32525997delins[NC_000012.11:g.qter_47519275]; NC_000023.10:g.pter_32526002delins[NC_000012.11:g.qter_47519198]

(b) Graphical representation of the translocation variant.

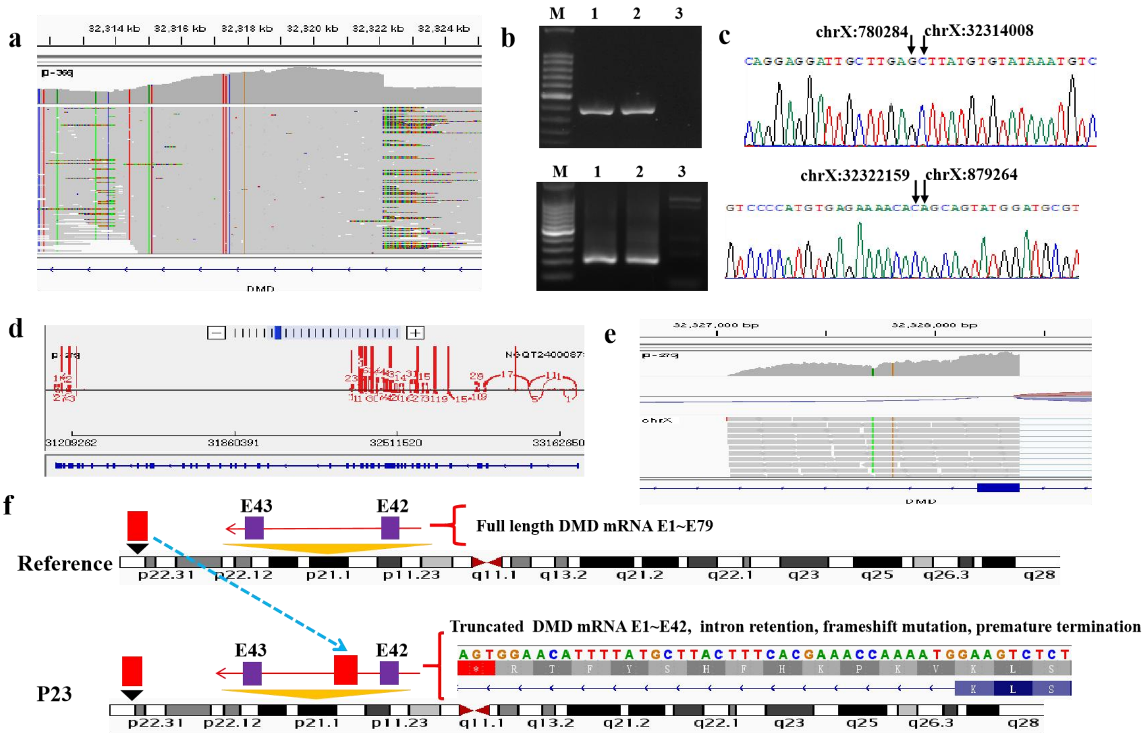

Fig. S20 Characterization of the insertion variant in P23.

(a) Long-read sequencing of the genomic DNA of P23 showed an insertion variant (g.32314008_32322159ins780284_879264; g.32314008_32322159dup). **(b)** PCR products spanning the insertion breakpoints showed an expected fragment in P23 and his mother (lanes 1 – 2), absent in a healthy control (lanes 3). M, size marker. **(c)** Sanger sequencing of the PCR products confirmed the insertion variant. **(d)** Sashimi plot visualization showed transcription termination in P23. **(e)** RNA sequencing showed transcription termination was caused by this variant. **(f)** Graphical representation of the insertion variant.

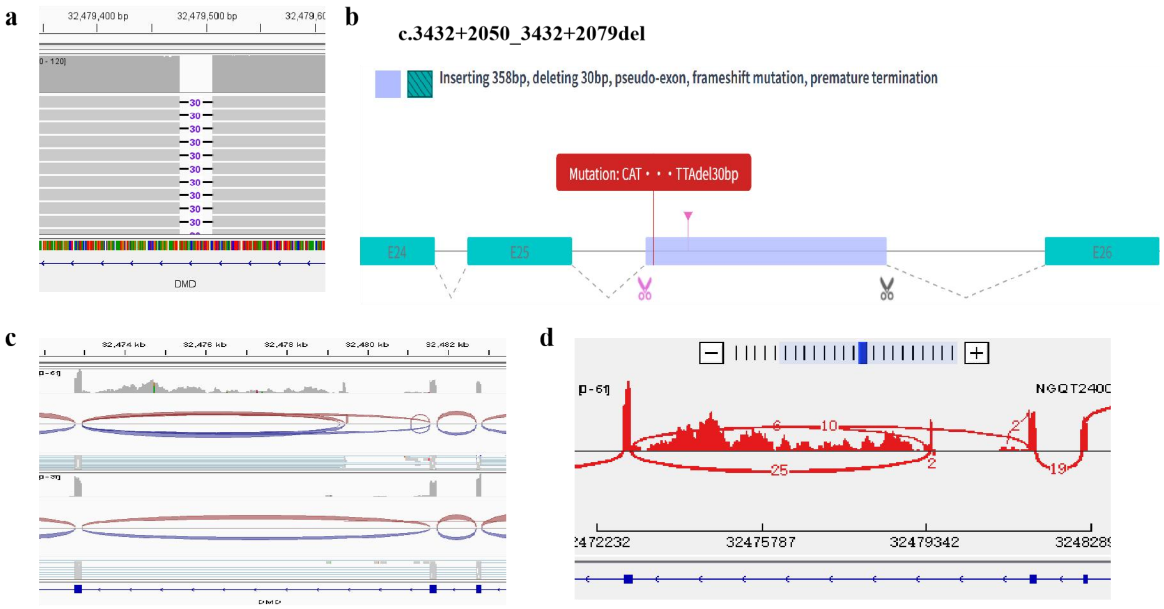

Fig. S21 Characterization of the deletion variant c.3432+2050_3432+2079del causing pseudoexon inclusion.

(a) Long-read sequencing of the genomic DNA of P30 revealed a deep intronic deletion variant (chrX:g.32479477_32479506del; c.3432+2050_3432+2079del).

(b) The online RNA Splicer tool predicted an aberrant splicing pattern caused by the deep intronic variant of c.3432+2050_3432+2079del.

(c) RNA sequencing showed that the variant caused pseudoexon inclusion (upper panel), compared to the healthy control (lower panel).

(d) Sashimi plot visualization showed a pseudoexon was caused by the variant.
